# IBD Patients Could Be Silent Carriers for Novel Coronavirus and Less Prone to its Severe Adverse Events: True or False?

**DOI:** 10.22074/cellj.2020.7603

**Published:** 2020-09-08

**Authors:** Shaghayegh Baradaran Ghavami, Shabnam Shahrokh, Nikoo Hossein-Khannazer, Anastasia Shpichka, Hamid Asadzadeh Aghdaei, Peter Timashev, Massoud Vosough

**Affiliations:** 1. Basic and Molecular Epidemiology of Gastrointestinal Disorders Research Center, Research Institute for Gastroenterology and Liver Diseases, Shahid Beheshti University of Medical Sciences, Tehran, Iran; 2. Gastroenterology and Liver Diseases Research Center, Research Institute for Gastroenterology and Liver Diseases, Shahid Beheshti University of Medical Sciences, Tehran, Iran; 3. Department of Immunology, School of Medicine, Shahid Beheshti University of Medical Sciences, Tehran, Iran; 4. Institute for Regenerative Medicine, Sechenov University, Moscow, Russia; 5. Department of Chemistry, Lomonosov Moscow State University, Moscow, Russia; 6. Department of Regenerative Medicine, Cell Science Research Center, Royan Institute for Stem Cell Biology and Technology, ACECR, Tehran, Iran

**Keywords:** Anti-TNF-α, COVID-19, Crohn’s Disease, IBD, Ulcerative Colitis

## Abstract

Inflammatory bowel diseases (IBDs) are chronic disorders of the gastrointestinal tract. The goal of IBD treatment is to reduce the inflammation period and induce long-term remission. Use of anti-inflammatory drugs including corticosteroids, immunosuppressants and biologicals, is often the first step in the treatment of IBD. Therefore, IBD patients in pandemic of infectious diseases are considered a high-risk group. The public believes that IBD patients are at a higher risk in the current coronavirus 2 pandemic. Nevertheless, these patients may experience mild or moderate complications compared to healthy people. This might be because of particular anti-TNF-α treatment or any immunosuppressant that IBD patients receive. Moreover, these patients might be silent carrier for the virus.

## Introduction

Inflammatory bowel diseases (IBDs) are chronic inflammatory disorders of the gastrointestinal tract; IBDs are categorized as Crohn’s disease (CD) and ulcerative colitis (UC) (1). The variation in the gut microbiota and certain genetic backgrounds as well as particular lifestyles are suggested as the main reasons for initiation and progression of these diseases (2). IBDs include two clinical stages, flare-up and remission, and the main therapeutic measures involve establishment and extension of the remission phase and avoiding flare-up occurrence (3). The medications used for management of the condition, are 5-aminosalycilic acid (5-ASA), immunosuppressants and biologicals targeting the immune system (1). Therefore, IBD patients are considered a high-risk group in epidemic and pandemic of infectious diseases.

Interestingly, in the recent pandemic of coronavirus disease (COVID-19), and the SARS-CoV epidemic in 2003, while the fecal samples of these patients were positive for the virus, they did not present any severe respiratory distress syndrome (4). In patients infected with SARS-CoV-2, the gastrointestinal symptoms such as diarrhea and nausea are more common compared to SARS-CoV patients. In addition, it was reported that while the SARS-CoV-2 test is negative for upper respiratory samples, stool samples remain positive for a few weeks after treatment (5). Remarkably, the angiotensin-converting enzyme-2 (ACE2) is the receptor for SARS-CoV-2 and it is expressed in different organs including the lungs, testis and ileum. This protein is also expressed on gut epithelial cells and secreted to the gut lumen (6).

The following two questions should be addressed in this context. First, whether IBD patients show mild or moderate signs and symptoms of COVID-19 compared to the others? Second, in the recent pandemic situation, could IBD patients be considered "silent carriers" and might they increase the disease spread rate?

The innate immune system has a crucial role in protecting body during viral infections. The innate immune system produces and releases interferon α (IFN-α), an essential cytokine that interferes with the viral replication, virulence and spread in the host during the early phases (7). One of the important activities of coronavirus 2 is suppression of transcription and secretion of IFN-α (8). Moreover, it was shown that, SARS-CoV-2 can block the antiviral effects of IFN-α, in vitro. This is an essential mechanism for coronavirus 2 to escape the host innate immune system (9).

Recently, it was considered that there is a regulatory cross-talk between IFN-α and tumor necrosis factor (TNF-α). Of note, this cross-talk was reported between IFN-α and anti-TNF-α biologicals in clinic. Banchereau et al. and Palucka et al. showed that when rheumatoid arthritis patients were treated with anti-TNF-α biologicals (infliximab, adalimumab, and certolizumab pegol), the expression of IFN-α-regulated genes was increased in the peripheral blood mononuclear cell (PBMC) compared to the control group. Moreover, it was shown that when the immune cells produce higher amounts of IFN-α, they are less prone to be infected with SARS-CoV-2 and other viruses (10). Therefore, in patients with IBD who were treated with anti-TNF-α, there might be an increase in the production of IFN-α against viral infections. This could justify why IBD patients who were infected with SARSCoV- 2 , could probably present less severe symptoms compared to others (11). Besides, in IBD patients, particularly those who are under anti-TNF-α treatment, the host innate immune system interferes more efficiently with viral replication cycle and the clinical presentations are more moderate (9, 12).

ACE2 regulates the renin–angiotensin system (RAS) by cleaving several peptides (Ang1-7). This biological activity limits inflammation reflecting a protective role of the ACE2-MasR pathway (13). ACE2 is expressed on the epithelial cells in different organs including lungs, kidneys, liver, brain, blood vessels and particularly, on the cell membrane of the gut and illume epithelial cells (14). ACE2 is known as the main receptor for spike (S) protein of SARS-CoV-2 and is crucial for colonization and entry of the virus into the target cells. The soluble form of ACE2 (sACE2) can act as a decoy molecule and cover the S protein on the virions and block colonization. This can prevent the successful binding of the viral particles to the surface of the cells (15). Remarkably, patients with active UC and CD have higher numbers of ACE2 in their affected tissues (16). Since IBD patients are 3 times more prone to viral infections such as CMV, EBV, varicella zoster virus, and HSV, possible correlations between immunosuppressive therapy/biological treatments and coronavirus infection in IBD patients, should be assessed (17).

It was reported that ACE2 could be a potential target for therapeutic protocols against COVID-19. Therefore, human recombinant soluble ACE2 (hrACE2) could be considered a novel candidate for new treatment strategies (15, 18). Additionally, in two studies, it was shown that soluble ACE2 could block SARS coronavirus 2 replication (4, 19).

RAS and ACE2 are key players in human IBDs (19). It was reported that upregulation of Ang I-VII and ACE2 might be a compensatory response to intestinal inflammation which could result in increased concentrations of circulating ACE2. In fact, in IBD patients, the soluble isoform of ACE2 is found at higher levels in the peripheral blood, compared to normal individuals (11, 20). Interestingly, the expression of ACE2 and Ang (1-7) are increased in terminal ileum and colon in CD and UC patients (21). Though, IBD patients are quarantined and protected well during the pandemic, they could be “silent carrier” of the coronavirus.

TNF-α converting enzyme (TACE) is a protease enzyme that splits ACE2 molecules from the surface of cells (22). Blockage of TNF-α pathway induces TACE activity and increases sACE2 level. Further, it was suggested that TACE might be a potential target for antiviral compounds used for treatment of COVID-19 patients (23). Upregulation of TACE activity increases detachment of the ACE2 form the surface of epithelial cells. Blocking TNF-α by monoclonal antibodies (mAbs) may result in increased TACE activity and higher rate of cleavage of ACE2 from the surface (24). This was observed in IBD patients who are receiving different treatments such as immunosuppressants, corticosteroids and biological medications, for preventing relapse (25).

**Fig.1:**
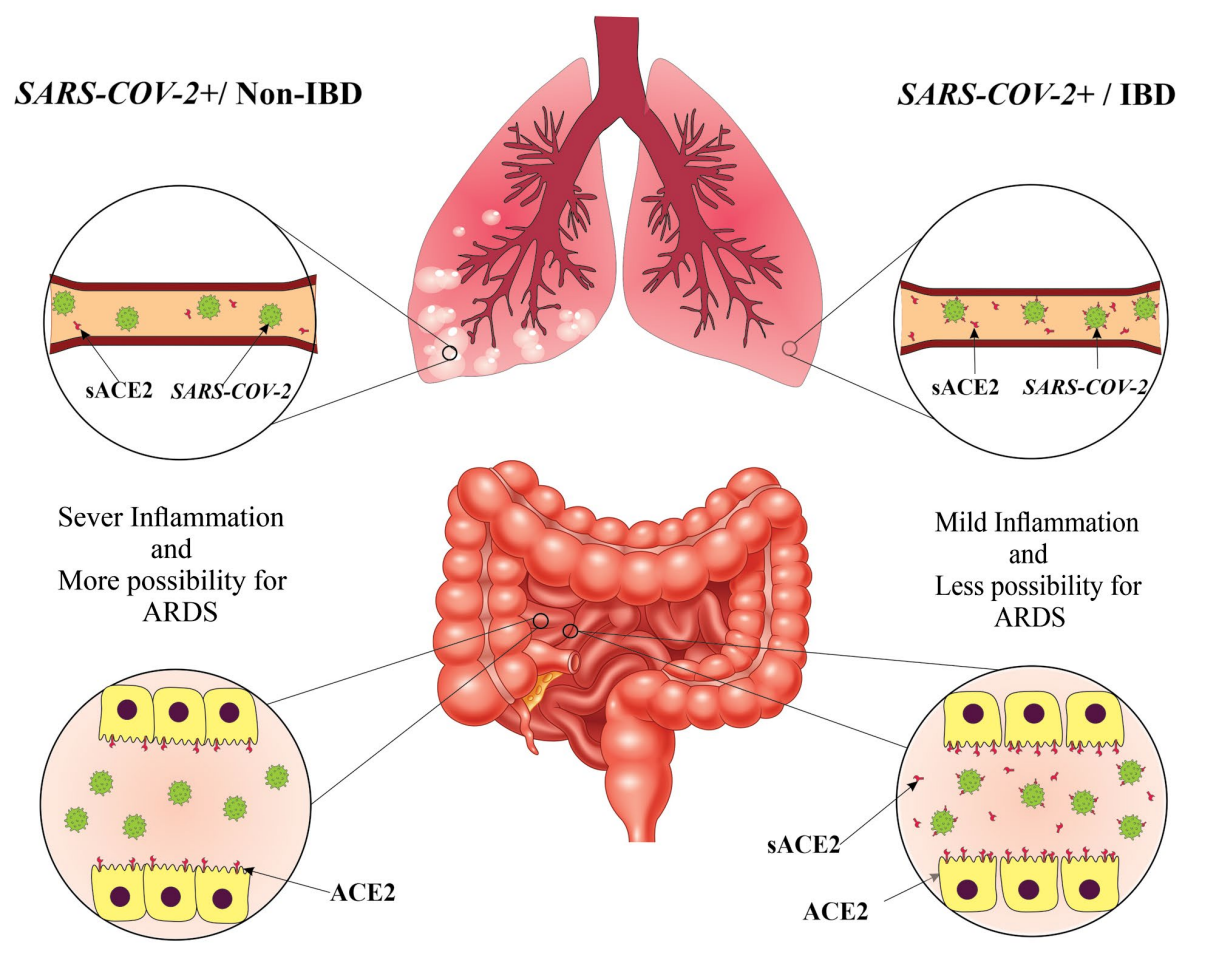
Schematic representation of ACE2 and sACE2 on epithelial cells and in blood circulation respectively in IBD and non-IBD patients. IBD; Inflammatory bowel disease, ARDS; Acute respiratory distress syndrome, and ACE2; Angiotensin-converting enzyme-2.

The main cause of acute respiratory distress syndrome (ARDS) in COVID-19 patients is the cytokine storm. The severity of COVID-19 is associated with increasing serum levels of IL-6, IL-7, IL-8, granulocyte-colony stimulating factor (G-CSF), IFN-γ, macrophage inflammatory protein 1-α (MIP1-α), and TNF-α. This situation increases recruitment of the immune cells into the lungs resulting in hyper inflammation in the patients and increasing adverse events and mortality (26). Accumulating evidence revealed that anti-inflammatory treatments could control ARDS (27). A multicenter, randomized controlled trial, approved the use of tocilizumab (an IL-6 receptor blocker licensed for cytokine release syndrome), in patients with COVID-19 pneumonia. Additionally, Janus kinase (JAK) inhibition could modulate both inflammation and viral entry into the cells in COVID-19 infection (28). Interestingly, IBD patients who regularly take cytokine blockers and immunosuppressant could control cytokine storm and the other related adverse events (11). Based on the specific type of treatment employed for each IBD patient, the severity of inflammation in the lungs and the antiviral immune responses may vary. This might help IBD patients in combating COVID-19 infection though the immune suppression can increase the risk of certain viral infections (29). It should be pointed out that further studies are required in this highly dynamic situation. There is no convincing evidence recommending that patients with IBD should stop their IBD-related medications (30). Nevertheless, elder IBD patients suffering from other comorbidities like obstructive lung diseases, diabetes mellitus, coronary heart diseases and hypertension might have an increased risk of COVID-19 (11). Figure1 schematically depicts this hypothesis.

Though the public believes that IBD patients are at a higher risk for SARS-CoV-2 infection and complications, these patients may experience mild or moderate complications compared to healthy people. However, these patients might be silent carrier for the virus and should maintain their social distancing more strictly. These statements explain our hypothesis that in IBD patients infected with SARS-CoV-2, less severe clinical complications and lower morbidity and mortality rates, might be observed.
